# Simultaneous Resection of Esophageal Carcinosarcoma with Cancer of the Stomach and Transverse Colon: A Case Report

**DOI:** 10.70352/scrj.cr.25-0130

**Published:** 2025-08-07

**Authors:** Kenichi Mizunuma, Masato Suzuoki, Ryo Takahashi, Shinya Otsuka, Hiroki Niwa, Hideyuki Wada, Kei Hiraoka, Kazuteru Komuro, Noriko Kimura, Nozomu Iwashiro, Masanori Ohara, Satoshi Hirano

**Affiliations:** 1 Department of Surgery, NHO Hakodate Medical Center, Hakodate, Hokkaido, Japan; 2 Department of Pathology, NHO Hakodate Medical Center, Hakodate, Hokkaido, Japan; 3 Department of Gastroenterological Surgery II, Hokkaido University Faculty of Medicine, Sapporo, Hokkaido, Japan

**Keywords:** esophagus, carcinosarcoma, gastric carcinoma, colon carcinoma, multiple cancers, simultaneous resection

## Abstract

**INTRODUCTION:**

Esophageal carcinosarcoma is a rare malignant neoplasm composed of both carcinoma and sarcoma components. Here, we report a case of esophageal carcinosarcoma in a patient with cancers of the transverse colon and stomach who underwent simultaneous resection of all 3 malignancies.

**CASE PRESENTATION:**

A 71-year-old man presented with dysphagia and was diagnosed with carcinoma in the mid-thoracic esophagus. Laboratory data showed normal tumor markers—carcinoembryonic antigen (CEA), squamous cell carcinoma antigen (SCC), and carbohydrate antigen 19-9 (CA19-9). Esophagoscopy confirmed the presence of a polypoid tumor in the middle thoracic esophagus. Endoscopic biopsy specimens were reported as spindle cell carcinoma. Colonoscopy revealed a semicircular tumor in the transverse colon. Simultaneous esophagectomy and regional lymphadenectomy and transverse colon resection using video-assisted thoracoscopic surgery (VATS) and hand-assisted laparoscopic surgery (HALS) were planned. Intraoperative findings revealed advanced cancer in the upper part of the stomach, which was also resected. Macroscopically, there was an 11-cm type 1 tumor in the middle thoracic esophagus and a 3.5-cm type 3 tumor in the gastric cardia. Microscopically, the esophageal tumor was composed of well-differentiated squamous cell carcinoma and spindle-shaped cells resembling leiomyosarcoma and was diagnosed as carcinosarcoma. Spindle-shaped sarcomatous cells were positive for vimentin and α-smooth muscle actin (αSMA) by immunohistochemistry. Most of the tumor showed highly atypical sarcomatous component that reached deep into the submucosa, and regional lymph node metastases were observed. Gastric cancer was a moderately differentiated tubular adenocarcinoma that penetrated the serosa and reached the peritoneal cavity. The transverse colon tumor was a well-differentiated adenocarcinoma invading the muscularis mucosa.

**CONCLUSIONS:**

We report a rare case of triple cancer, including esophageal carcinosarcoma. Simultaneous resection of esophageal carcinosarcoma with multiple cancers should be carefully considered based on the patient’s condition and reconstruction method.

## Abbreviations

CA19-9
: carbohydrate antigen 19-9
CEA
: carcinoembryonic antigen
CK7
: cytokeratin 7
FDG-PET
: fluorodeoxyglucose positron emission tomography
HALS
: hand-assisted laparoscopic surgery
SCC
: squamous cell carcinoma antigen
VATS
: video-assisted thoracoscopic surgery
VC
: vital capacity
αSMA
: α-smooth muscle actin

## INTRODUCTION

Carcinosarcoma of the esophagus is a rare malignant neoplasm comprising both carcinoma and sarcoma components. Among esophageal tumors, the frequency of carcinosarcoma is approximately 2%,^1)^ and the simultaneous development of multiple cancers is even rare. Herein, we report a patient with esophageal carcinosarcoma and transverse colon and gastric cancers who underwent synchronous VATS and HALS.

## CASE PRESENTATION

A 71-year-old man with dysphagia was admitted to our hospital and underwent an upper gastrointestinal endoscopy. There was esophageal stenosis caused by an esophageal tumor. The patient had no significant medical or family history. He drank 350 mL of liquor daily and smoked 1 pack of cigarettes per day for more than 50 years. Laboratory data showed normal tumor makers—CEA, SCC, and CA19-9, and high inflammatory response with white blood cell count of 13800/µL and C-reactive protein level of 12.6 mg/dL. Spirometry indicated restrictive ventilatory impairment: VC of 2.0 L and %VC of 57.9%.

**Fig. 1:**
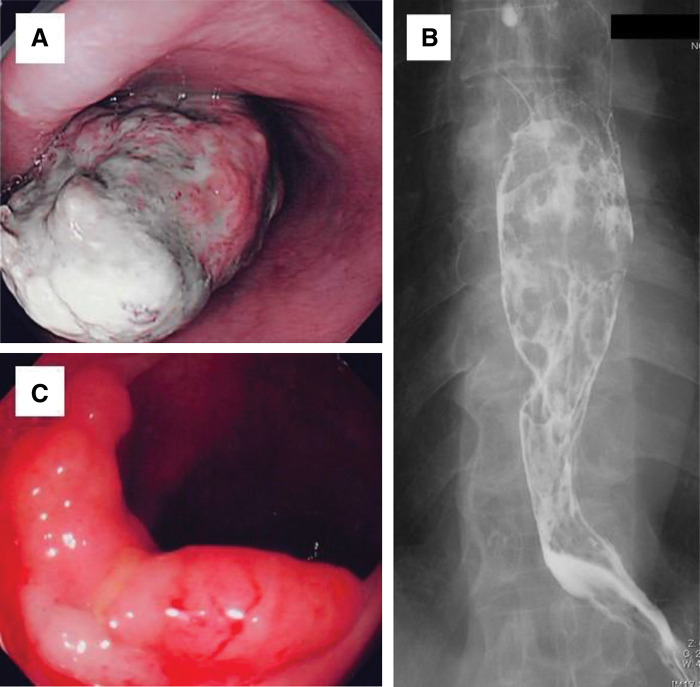
Esophagoscopy and colonoscopy findings (**A**) Esophageal endoscopy revealed a polypoidal type 1 tumor in the midthoracic esophagus. The surface of the tumor was covered with white belag. (**B**) Esophagogram showed an intraluminal filling defect in the mid-thoracic esophagus. (**C**) Colonoscopy confirmed a laterally spreading semicircular tumors in the transverse colon.

Esophagoscopy confirmed the presence of a polypoid tumor in the middle thoracic esophagus. The surface of the tumor was covered with belag (**Fig. 1A**). Endoscopic biopsy specimens were reported as spindle cell carcinomas. We could not observe the total size of the tumor, the lower esophagus, or the upper stomach because of this bulky tumor in the middle esophagus. Upper gastrointestinal barium study revealed an intraluminal filling defect in the middle intrathoracic esophagus (**Fig. 1B**). Computed tomography showed a polypoid tumor in the middle intrathoracic esophagus that did not invade other organs or the enlarged lymph nodes. Colonoscopy revealed a semicircular tumor in the transverse colon (**Fig. 1C**). Endoscopic biopsy specimens showed tubular adenomas with moderate atypia. However, the tumor was too large to undergo endoscopic mucosal resection and was most likely a carcinoma. We planned to resect the esophageal cancer and colon tumor simultaneously and perform VATS and HALS esophagectomy and regional lymphadenectomy with gastric conduit reconstruction. However, intraoperatively, an advanced cancer was identified in the upper part of the stomach, and the gastric cancer was also resected simultaneously. The upper stomach cancer was resected after confirming that the margins were negative and at a distance from the tumor. A gastric conduit was created with the remnant stomach. The length of the gastric conduit was sufficient, and a left cervical gastric conduit and esophageal anastomosis was performed. We performed a transverse colon resection and reconstructed the colonic anastomosis. The operative time was 4 h 26 min, and the estimated blood loss was 104 mL.

**Fig. 2:**
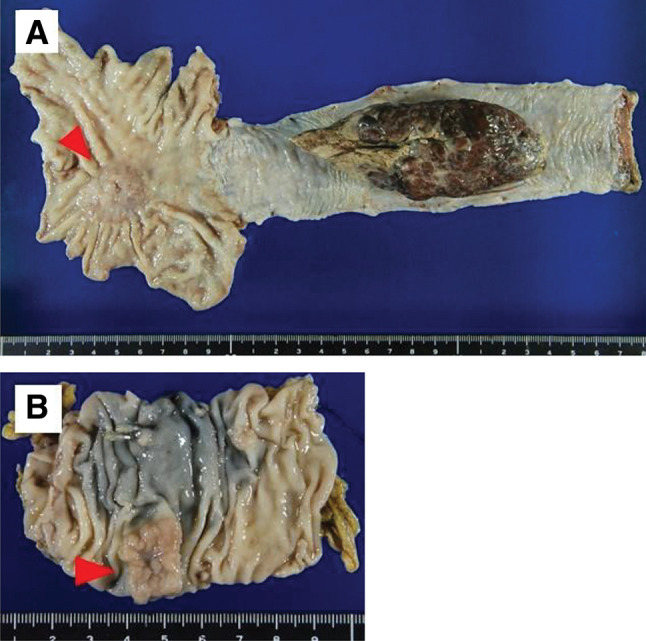
Macroscopic findings of the resected specimen (**A**) A type 1 esophageal tumor, 11 cm in size, was seen in the middle thoracic esophagus, and a type 3 gastric tumor (3.5 cm) was located in the cardia of the stomach (arrowhead). (**B**) The transverse colon showed a 1.5 cm laterally spreading tumor (arrowhead).

**Fig. 3:**
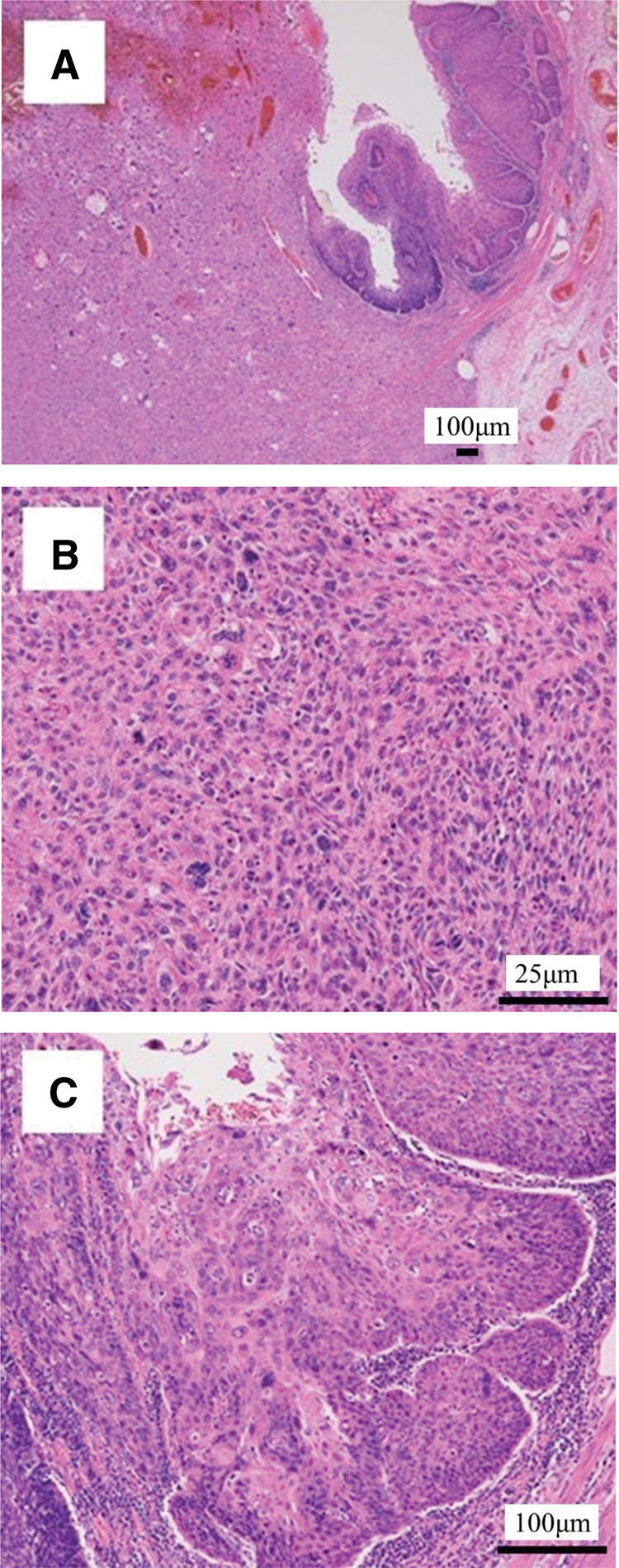
Microscopic findings of the esophageal tumor (**A**) The esophageal tumor comprised well-differentiated spindle-shaped cells and squamous cell carcinoma components (hematoxylin-eosin staining). (**B**) The proliferating spindle cells of the sarcoma intermingled. Numerous mitotic figures were observed. (**C**) The mucosal side of the esophageal tumor showed a well-differentiated squamous cell carcinoma component.

**Fig. 4:**
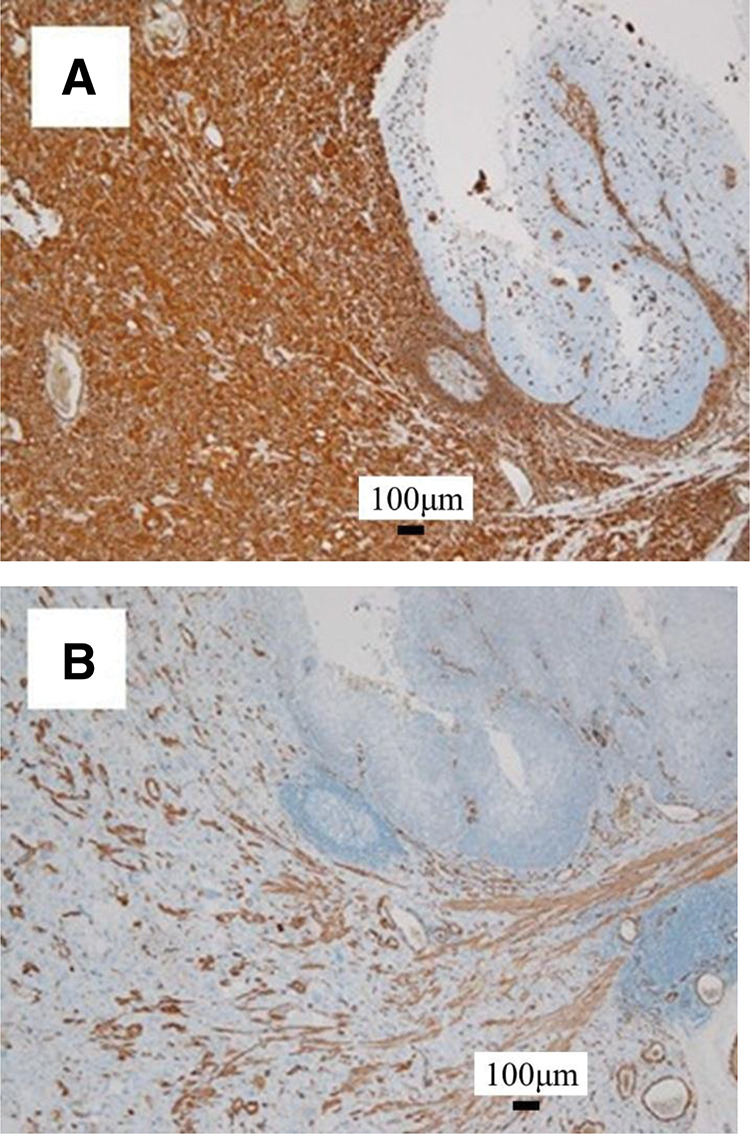
Histopathological immunohistochemical findings (**A**) Vimentin was positive in the spindle cells but negative in the squamous cell carcinoma cells. (**B**) Similarly, αSMA was positive in the spindle cells but negative in the squamous cell carcinoma cells. αSMA, α-smooth muscle actin

Macroscopically, there was an 11-cm type 1 tumor in the middle thoracic esophagus, and a 3.5-cm type 3 tumor in the cardia of the stomach (**Fig. 2A**). The transverse colon showed a 1.5 cm lateral spreading tumor (**Fig. 2B**). Microscopically, the esophageal tumor consisted of spindle cells and squamous cell carcinoma component (**Fig. 3A**). Most of the tumor showed spindle cells extending deep into the submucosa (**Fig. 3B**). By contrast, the tumor in the luminal surface of the esophageal mucosa showed a highly differentiated squamous cell carcinoma that had infiltrated the muscularis mucosa (**Fig. 3C**). Gastric cancer was a moderately differentiated tubular adenocarcinoma that invaded the serosal surface. The tumor in the transverse colon was a well-differentiated adenocarcinoma that had invaded the muscularis mucosa. Immunohistochemically, spindle cells were positive for vimentin and αSMA (**Fig. 4A** and **4B**). The squamous cell carcinoma cells were positive for CK7, p40, p63, AE1/AE3, and CAM5.2 and negative for vimentin and αSMA (**Fig. 4A** and **4B**).

Cancer was found in 2 right paracardial lymph nodes. One of them showed SCC, and the other showed adenocarcinoma. SCC metastasis was observed in the lymph nodes of the left recurrent nerve and middle thoracic paraesophageal lymph nodes.

The patient was finally diagnosed with esophageal carcinosarcoma classified as T1bN2M0 Stage IIIA, gastric cancer staged as T4a N0 M0 Stage IIB, and transverse colon cancer staged as T1 N0 M0 Stage I (UICC-TNM 8th). The patient’s postoperative course was uneventful. After discharge, the patient received adjuvant chemotherapy and remained free of disease after 1 year of follow-up.

## DISCUSSION

Esophageal carcinosarcoma is a relatively rare tumor, accounting for 0.5%–2.8% of all esophageal malignancies, and like other esophageal cancers, it occurs most frequently in the mid-thoracic esophagus.^1–3)^ Tumors often present as polypoid lesions, occupying the lumen of the esophagus and causing obstruction, and are easily detected at a relatively early stage. Carcinosarcoma is associated with a high risk of lymph node metastasis, even if it is detected at an early stage. In the present case, carcinosarcoma caused obstruction, leading to dysphagia and regional lymph node metastasis was present.

The treatment of esophageal carcinosarcoma is usually esophagectomy and lymph node dissection, if radical resection is possible, which is the basis for long-term survival.^4)^ Similar to conventional esophageal cancers, chemoradiotherapy may be an option for patients with advanced malignancies. Kobayashi et al.^5)^ reported 4 cases of esophageal carcinosarcoma treated by chemoradiotherapy. In 3 cases, the tumor size decreased, and in 1 case showed a complete response of the primary tumor. All patients underwent surgery after chemoradiotherapy, and the short-term results were relatively good. Neoadjuvant chemotherapy is the preferred treatment for advanced tumors. Katsuya et al.^6)^ summarized the clinical characteristics of preoperative chemotherapy for esophageal carcinosarcoma. They found no significant difference in overall survival between 6 patients treated with neoadjuvant chemotherapy and 13 patients treated with surgery. In our case, we performed an esophagectomy because the esophageal tumor had not invaded other organs and was considered resectable. The colon tumor was also suspected to be curable by simultaneous resection. The gastric cancer could not be accurately diagnosed preoperatively; therefore, the treatment plan had to be discussed intraoperatively.

When esophageal cancer is accompanied by gastric cancer, the accuracy of the diagnosis of gastric cancer influences the treatment strategy. In the absence of an accurate diagnosis of gastric cancer, total gastrectomy and lymph node dissection, followed by intestinal or colorectal reconstruction, may have been the optimal treatments. When resecting cancers of the upper gastric body and cardia, if more than half of the stomach can be spared and there are no lymph node metastases, proximal gastrectomy is considered, unlike standard surgery.^7)^ In our case, preoperative CT tomography revealed no regional lymph node metastasis. The intestinal or colorectal tract is associated with a higher incidence of gangrene than the gastric conduit.^8)^ When reconstruction of the intestinal or colorectal tract is performed, the anastomotic site increases, which requires more operating time and is more invasive for the patient. In our patient, intraoperatively, the tumor was in the upper part of the stomach and the rest of the stomach could be preserved. To reduce the invasive procedures, we resected the esophagus and upper stomach and reconstructed the gastric conduit. Intraoperative assessment indicated that the gastric cancer may be advanced. However, the usefulness of proximal gastrectomy in advanced cancer has been reported,^9,10)^ and it may be considered if the resection margin from the tumor can be secured. A surgical margin of 3 to 5 cm is recommended, depending on the invasive pattern of the tumor.^7)^ In our case, the margins were close together but were negative for tumor. Accurate preoperative diagnosis of gastric cancer is necessary. The limitation of the upper endoscopy in detecting gastric cancer in a patient with an obstructing esophageal cancer has been pointed out.^11)^ Therefore, when there is a large tumor occupying the lumen, other testing methods such as FDG-PET are recommended. In this case, FDG-PET was considered but was not performed due to logistical constraints and the urgency of surgical planning.

Carcinosarcoma of the esophagus presenting along with multiple cancers is extremely rare. There have only been reports of carcinosarcoma and gastric cancer,^12)^ and there have been no reports of 3 different cancers as in this case. One case report described 3 carcinosarcomas simultaneously found in the esophagus that were treated surgically.^13)^ Carcinosarcoma requires careful surveillance, and preoperative imaging is essential because early cancer diagnosis benefits the patient. Lynch syndrome is a common inherited colorectal cancer syndrome caused by germline mutations in DNA mismatch repair genes. Gastric cancer is commonly associated with colorectal cancer, and a few cases of esophageal cancer have been observed, although the genetic predisposition is unknown.^14)^ Although the lack of molecular biological testing precluded the diagnosis of Lynch syndrome, it may be important to consider a hereditary cancer syndrome in a patient with multiple gastrointestinal malignancies.

When considering the treatment of this case, we referred to previous reports of multiple cancers associated with esophageal cancer, and treated the esophageal carcinosarcoma using the conventional treatment for esophageal cancer. Papaconstantinou et al.^15)^ reviewed simultaneous esophageal cancer resection. Seventy-five patients (71%) had a second primary neoplasm located in the stomach, accounting for the majority of the cases in the entire cohort, and only 2 patients (1%) had a second primary colorectal malignancy. In these patients, the synchronous procedure is not curative and may have been feasible with acceptable safety. Esophageal carcinosarcoma can grow into a large polypoid tumor, which may prevent adequate visualization of the esophageal lumen and stomach during preoperative examination. In addition, even a small number of gastric cancers can complicate the procedure; therefore, intraoperative observation and resection should be considered, assuming that the area that could not be observed by the upper endoscope also contains tumors. These findings suggest that synchronous surgery may not affect cancer prognosis; however, the surgical technique should be considered.

Although there are a few reported cases of triple cancer resection, including esophageal cancer, they are cases of 2-stage surgical treatments. Akiyama et al.^16)^ reported 2-stage operations for triple cancers of the esophagus, colon, and liver. They had performed sigmoid colectomy and liver resection initially, followed by esophagectomy and reconstruction, taking into account the advanced stage of colon and liver cancer. Fukaya et al.^17)^ also resected synchronous triple cancers of the esophagus, stomach, and ampulla of Vater in 2-stage operations. They performed only esophagectomy first, followed by total gastrectomy and pancreatoduodenemtomy; esophageal reconstruction was achieved using the ileocolon as a second operation. In each case, the cancers were resected in 2 stages because esophagectomy is a highly invasive procedure that can cause complications in patients. The timing of esophagectomy differed depending on the degree of cancer progression and the invasiveness of the surgery. We chose to perform a 1-stage surgery based on the patient’s good performance status, early-stage transverse colon cancer, and the possibility of gastrointestinal tube reconstruction. When esophagectomy is performed in a patient with esophageal cancer and multiple gastrointestinal cancers, it is important to consider the surgical approach and reconstructive organs.

Surgery for multiple cancers of the esophagus requires consideration of not only the invasiveness of the procedures but also the method of reconstruction. In synchronous esophagectomy and gastrectomy, reconstructive organs vary depending on the location of the gastric cancer. Several reports have described the colonic interposition technique when gastrointestinal reconstruction cannot be performed due to gastrectomy.^18,19)^ Furthermore, if colonic resection is necessary, the reconstruction site depends on the location of the colorectal tumor. If simultaneous resection is not possible or colorectal interposition is necessary, 2-stage surgery must be considered. In our case, we reconstructed the gastric conduit and resected the colorectal cancer using HALS because the gastric cancer was located in the upper part of the gastric body, minimizing the need for gastrectomy. Owing to the HALS method of laparotomy in the upper abdomen, transverse colon cancer in the mid-colon could be treated. HALS has technical feasibility and no significant difference in mortality and morbidity compared with laparoscopic surgery in esophagectomy.^20)^

In our case, simultaneous esophageal surgery did not cause any complications or affect the postoperative course; however, 2-stage surgery might be an option depending on the progression and location of the gastric or colonic cancer.

## CONCLUSIONS

Herein, we reported a case of triple cancer, including carcinosarcoma of the esophagus. Therefore, it is important to identify any other cancers preoperatively in patients with carcinosarcoma. Simultaneous resection of the 3 malignant tumors, including carcinosarcoma, is possible, but the procedure should be carefully considered based on the patient’s condition and the reconstruction method.

## ACKNOWLEDGMENTS

We would like to thank Editage (http://www.editage.jp) for English language editing.

## DECLARATIONS

### Funding

No funds, grants, or other support was received.

### Authors’ contributions

KM drafted the manuscript.

MS and SH contributed to revise the manuscript.

NK contributed to the pathological diagnosis.

RT, SO, HN, HW, KH, KK, NI, and MO helped care for the patient and draft the manuscript.

All authors read and approved the final manuscript.

### Availability of data and materials

Not applicable.

### Ethics approval and consent to participate

Not applicable.

### Consent for publication

Informed consent was obtained from the patient for the publication of this case report.

### Competing interests

Kei Hiraoka received advisory fees from Takeda Pharmaceuticals. The other authors declare that they have no competing interests.
